# Application of CRISPR/Cas9 to human-induced pluripotent stem cells: from gene editing to drug discovery

**DOI:** 10.1186/s40246-020-00276-2

**Published:** 2020-06-26

**Authors:** Claudia De Masi, Paola Spitalieri, Michela Murdocca, Giuseppe Novelli, Federica Sangiuolo

**Affiliations:** Department of Biomedicine and Prevention, University of Rome “Tor Vergata”, Rome, Italy

**Keywords:** Human-induced pluripotent stem cells (hiPSCs), CRISPR/Cas9, Gene editing, Drug discovery, HIV and SARS-Cov-2 infection, Gene therapy

## Abstract

Human-induced pluripotent stem cells (hiPSCs) and CRISPR/Cas9 gene editing system represent two instruments of basic and translational research, which both allow to acquire deep insight about the molecular bases of many diseases but also to develop pharmacological research.

This review is focused to draw up the latest technique of gene editing applied on hiPSCs, exploiting some of the genetic manipulation directed to the discovery of innovative therapeutic strategies. There are many expediencies provided by the use of hiPSCs, which can represent a disease model clinically relevant and predictive, with a great potential if associated to CRISPR/Cas9 technology, a gene editing tool powered by ease and precision never seen before.

Here, we describe the possible applications of CRISPR/Cas9 to hiPSCs: from drug development to drug screening and from gene therapy to the induction of the immunological response to specific virus infection, such as HIV and SARS-Cov-2.

## Background

Advances in medical research have always been characterized by the continuous exploration of resources able to improve and speed up the achievement of experiments designed to uncover the elements shaping a disease, with the final aim to pinpoint prognostic and diagnostic factors and above all, to identify therapeutic targets and drugs. Frequently, these aims can be reached combining crucial findings, which is the case featuring the conjunction of the latest gene editing techniques and the technology capable to induce pluripotency to somatic cells through the reprogramming mechanisms. Since their discovery, induced pluripotent stem cells (iPSCs) have proved to have a great potential, including the option to overcome ethical and safety concerns related to the use of embryonic stem cells [1]. To date, the chance to access to human cell lines through minimally invasive techniques, such as skin punch biopsy, hair, urine, or blood samples, but also the collection of chorionic villus and amniotic fluid samples, makes obtaining human iPSCs (hiPSCs) more feasible [2–5]. Therefore, today, these cells are used for extensive studies employing both donor-derived healthy and diseased cell lines. In fact, hiPSCs can exhibit phenotypes close to human pathology, so they can represent a disease model clinically relevant and, in some cases, more predictive than the currently available animal-derived or tumor cell-derived cells [6]. hiPSCs are able to reflect patient physiology, pathophysiology, and pharmacological responsiveness, imitating human organs and their microenvironment, particularly when cultured under conditions recapitulating tissue architecture in multicellular spheroids or organoids [7]. Taken together these features, in concomitance with the recent evidences about hiPSCs paracrine effects [8], make these cells the ideal candidate to promote endogenous regenerative repair or to replace injured tissues after cellular transplantation, maintaining patient’s genetic background and limiting immune rejections, with the intent to treat diseases in a more personalized manner [8, 9].

Recently, the application of hiPSCs has been frequently associated with the use of gene editing, targeting the disease-causing gene in order to study the pathophysiology even more deeply, carry out drug screening, and improve cell therapeutic potential [10].

Actually, the most powerful gene editing tool is embodied by clustered, regularly interspaced, short palindromic repeats (CRISPR) and CRISPR-associated protein (CRISPR/Cas9). This can be considered one of the most powerful and versatile technology both for gene editing and transcriptional control but also for epigenetic modulation. Its assembling with hiPSCs is having important implications for scientific research [11].

**Fig. 1:**
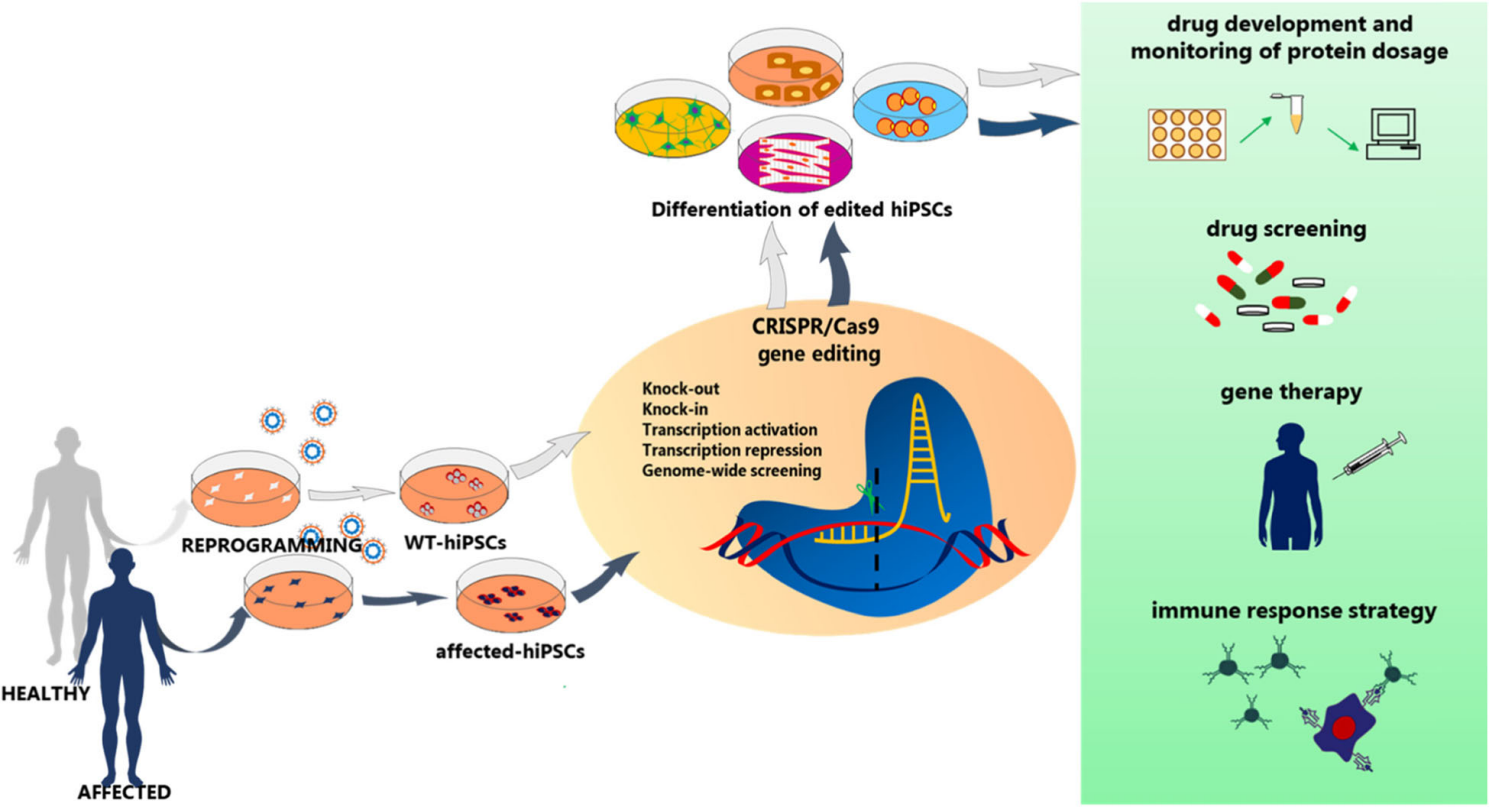
Workflow of the research involving hiPSCs and CRISPR/Cas9 gene editing for the investigation of new drugs and therapeutic alternatives

The aim of this review is to summarize the pivotal roles of hiPSCs in medical and pharmacological research in concomitance with the employment of CRISPR/Cas9 system, focusing the attention on their expediencies for the investigation of new drugs and therapeutic alternatives (Fig. 1).

## hiPSCs as a drug discovery device

Just after the spread of the reprogramming technology, getting a disease model through the employment of hiPSCs has been used to test several candidate drugs for different pathologies. This expedites the process leading to the clinical application of a particular compound, especially if it has been previously approved for the treatment of other diseases, improving the identification of possible targets and drugs, but also optimizing the selection and stratification of trial participants [11]. However, hiPSCs constitute a successful method when they have to reproduce monogenic diseases, while they may still have limitations in cases of sporadic disorders, where the environmental factors could contribute to the onset of a de novo mutation [9, 12]. Another challenge is embodied by the reproduction of late-onset disorders, since differentiated cells from hiPSCs have fetal-like properties, so it is necessary the induction of cellular aging [13, 14]. In addition, the epigenetics status of hiPSCs individual clones could be influenced by factors of culture conditions during the reprogramming process [12].

The chance to get hiPSCs isogenic cell line ensures to establish a genetically defined condition, overcoming the genetic background variations between patient and controls hiPSCs, as well as any other variables due to age or sex. In this way, a full range of controls is available to support (wild type (WT)-hiPSCs, patient-hiPSCs, and corrected-patient-hiPSCs) the validation of drug screening results [9, 10].

The first hiPSCs large-scale drug screening was performed in 2009, when patient-specific hiPSCs were employed to model familial dysautonomia (FD, OMIM #223900), a peripheral neuropathy coupled with the degeneration of the autonomic and sensory neurons, caused by a point mutation in IkB kinase complex-associated protein (IKBKAP8), resulting in tissue specific splicing defect. Lee and colleagues [15] found three important parameters of FD through hiPSCs disease model. Then, they tested some candidate drugs that could have had an impact on the features found, coming to discover that the kinetin plant hormone could mitigate the pathological phenotype [15].

This cellular approach has led to the evaluation of several compounds for severe diseases, and from then, many drug screening tests have been conducted on hiPSCs, both for testing the efficacy and the toxicity of different compounds.

Some of the latest hiPSCs drug screenings are summarize in Table 1.

**Table 1:** Latest hiPSCs drug screening

Research field	hiPSCs-derived cells	Drugs tested	Outcome	Ref
Colon cancer	Cardiomyocytes and endothelial cells, cocultured with tumor spheroids	2 anticancer drugs were tested in order to simultaneously assess cardiac toxicity and antitumor effects	Demonstration of the feasibility to simultaneously assess cardiac toxicity and antitumor drugs effects	[16]
Lesch-Nyhan disease (OMIM #300322)	Cortical neurons	Test of 3838 compounds	Identification of 6 pharmacological compounds correcting phosphoribosyltransferase (HGPRT) deficiency–associated neuronal phenotypes	[17]
Progressive fibrosis	Mesenchymal-like cells	Libraries of ~ 17000 small molecules	Identification of anti-fibrotic small molecule	[18]
Neuroprotective activity of pharmacological compounds	Embryoid bodies, neuronal precursors and neurons	Screening of peptides of the melanocortin family and endocannabinoids for cytotoxicity, embryotoxicity, and neuroprotective potential	Both melanocortin peptides and endocannabinoids exerted neuroprotective effects	[19]
Pro-regenerative drug development	Cardiomyocytes	Test of 150 small molecules with pro-regenerative potential	Identification of 2 pro-proliferative compounds acting via the mevalonate pathway.	[20]
Alzheimer’s disease (OMIM #104300)	Neurons	Test of R33 molecule	R33 is able to reduce Aβ and pTAU	[21]
Schizophrenia (SZ)	Neural progenitor cells	Screening of 135 drugs with predicted or known interactions in SZ-biology	Identification of 52 drugs ameliorating the SZ-related transcriptomic signature hiPSCs-derived neural progenitor cells	[22]
Amyotrophic lateral sclerosys (ALS) (OMIM #105400)	Motor neurons	1416 compounds involved in motor neurons survival	Identification of Src/c-Abl inhibitors increasing the survival rate of ALS motor neurons	[23]
Homozygous familial hypercholesterolemia (OMIM #144010)	Hepatocytes	Screening of a library of 2320 existing drugs	Identification of 5 cardiac glycosides able to reduce the hepatocytes production of apoB	[24]
Long-QT syndrome	Cardiomyocytes	Test of LUF7346, known hERG allosteric modulators	LUF7346 is able to rescue some phenotypic features of Long-QT-syndrome	[25]

Very recently, a trial has been started with the aim of testing the clinical efficacy of Bosutinib, a Src/c-Abl inhibitor [26], identified as a candidate molecular target therapy for amyotrophic lateral sclerosis (ALS, OMIM #105400). Src and Abl are tyrosine kinase associated with proliferation, apoptosis. and angiogenesis; they are considered a target of cancer therapy [23]. Different studies have demonstrated that some brain-penetrant tyrosine kinase inhibitors can attenuate the phenotype of neurodegenerative diseases associated with protein aggregation [27, 28]. Before developing the clinical trial, a drug phenotypic screening of existing Src/c-Abl inhibitors was performed using motor neurons generated from ALS patient-derived hiPSCs [23]. This advanced study resulted in a hiPSCs-based drug repurposing, as Bosutinib had already been approved by Food and Drug Administration (FDA) for the treatment of chronic myelogenous leukemia (CML, OMIM #608232) [26]. To date the mentioned trial is going to evaluate the efficacy, safety, and tolerability of Bosutinib in combination with the physical conditions of ALS patients, especially since this drug may cause some adverse effects [26, 29].

Actually, many efforts are being made to cut down the costs of drug development and to speed up this process, searching for novel tools that allow to predict drugs side effects, but also to test new drugs ensuring reliability and reproducibility in the outcome of drug treatment [30]. The ideal device should be provided with all of these features, without neglecting the genetic and epigenetic variations, but also the environmental factors and other situations that can potentially promote an increase in complexity level of pharmaceutical testing, such as the possible drug-drug interaction [6].

To meet these needs, the attention is now focused on the Organ-On-a-Chip (OOC) technology. This is a two-dimensional or three-dimensional microfluidic device of engineered biomaterials with an extracellular matrix amenable for the reproduction and observation of human cells behavior, from the adhesion to migration and from replication to differentiation [6]. The OOC consists of an array of microfluidic channels, which recurrently perfuse biological fluids that contain nutrients, biological factors and drugs, in a controllable manner [6]. In addition, it could be combined with an automated microdevice-monitoring component, which permits repeated measurements of the physiological parameters for the evaluation of drugs effects and toxicity. The OOC could be engineered using hiPSCs, offering the chance to build a personalized drug-testing platform, providing an effective uptake of the human pathophysiology, specifically designed for each patient [6]. Anyway, the OOC technology is characterized by some limitations including the technical challenges of the fabrication of the device but also the cost and inadequacy of acquired human materials. Furthermore, the device should provide a functional organ replication, so it is important to achieve an adequate growth and differentiation of the human tissues. This could represent a risk, because the use of particular growth factors and differentiation reagents, required for a particular cell type, could adversely affect other cells [6].

One of the strengths of the hiPSCs is based on their use not only for target-based screening but also for phenotypic screening. In the last few years, the phenotypic screenings are undergoing a reassessment to which hiPSCs have contributed, above all because they provide an easy access to multiple cell types disease-involved, especially those hard to obtain [31, 32]. For example, phenotypic screening for pain research has always been hampered by the lack of recruit enough neuronal cell types [33]. Now sensory neurons can be derived from hiPSCs, and they can be recruited to develop functional assay of neuronal excitability to be then employed for the evaluation of the phenotypic effects of a small-targeted validation compound subset [33]. Lately, hiPSCs have set the foundation for the development of a 3D human stem cell platform to screen cortical organoids with high-throughput screening (HTS) [34]. By assembling the reprogramming technology with the use of high content imaging, it was possible to obtain Serum-Free Embryoid Bodies (SFEBs) and then to determine neurite outgrowth and cellular composition. In this way, the detection of neurite morphology and multi-electrode array analysis were carried out [34]. This approach turned out to be a valid idea for countering the experimental variability common in 3D cultures; this study makes the conjunction between SFEBs and HTS the baseline for phenotypic drug screening [34].

## CRISPR/Cas9 system: the latest tool for gene editing

Gene editing acts as a tool to modify genome, allowing to correct, write, or remove a genetic information into a specific DNA sequence. Today, the available devices to edit DNA are provided with unprecedented ease and precision [35]. This can be translated into the possibility to repair the putative causative lesions in patient-derived cells or to introduce them in cells derived from healthy individuals, thus identifying the mutations involved in the disease phenotype [36].

From the 1970s to the 1980s, several methods were studied to alter a specific DNA locus, when genomic changes were carried out in yeast and in mice, using a gene target approach based on the process of the homologous recombination (HR) [37]. Recent advances have increased the efficiency of gene editing, implying the acquirement of the skill to induce a targeted DNA double-strand break (DSB) in the sequence of interest. Actually, the three main procedures able to induce DSBs are zinc finger nucleases (ZFNs), transcription activator-like effector nucleases (TALENs), and CRISPR/Cas9. They are all platforms defined by high target specificity, ease of use, and efficiency [10, 37]. Their activity is led by a nuclease, and their potential has been observed in natural biological processes [37].

ZFNs consist essentially of a set of a zinc finger DNA-binding domain, originally identified in sequence-specific eukaryotic transcription factors, fused with a DNA-cleavage domain from a bacterial protein [37]. They are designed to recognize 3–6 nucleotide triplets, with the frequent engagement of two or more zinc fingers [10, 38]. ZFNs-derived genomic modification is based on three main steps: the bond to the target sequence, the cleavage, and the genomic modification resulted from the activation of endogenous DNA repair [37]. These are the same passages exploited by TALENs, which are restriction enzymes secreted by plant pathogenic bacteria when they promote infection [10, 37]. A DNA-binding domain and a DNA-cleavage domain able to introduce DSBs into the DNA target sequence characterize TALENs technologies. The mechanism of action is based on a simple one-to-one code of recognition between modules in the protein and base pairs in the DNA target [10].

TALENs and ZFNs are broadly used both for disease modelling and therapeutic purposes, but their accuracy and specificity are less than those of CRISPR/Cas9. Moreover, there are still difficulties to face about the protein design, synthesis, and validation that do not allow their routine use [10, 37]. By the way, if a homologous donor DNA is provided, repair can proceed by HR. Otherwise, the break can be repaired by non homologous end joining (NHEJ) with the possibilities to introduce occasional errors, like small insertions and deletions (indels) [37, 39].

The CRISPR/Cas9 system was born to be a system of the adaptive immunity to viruses and plasmids in *Bacteria* and *Archaea*. It is known that three CRISPR/Cas9 types exist [40]. Among them, type II is the best known and finds application in genome engineering, because it requires only a single protein for RNA-guided DNA recognition and cleavage. Type II is a two-component RNA-programmable system, based on an endonuclease Cas9 that uses RNA guide sequences to form base pairs with DNA target sequences [40]. This recognition enables Cas9 to introduce a site-specific double-strand break in the DNA. The RNA-guide sequence is an RNA-duplex of tracrRNA (transactivating crRNA) and crRNA (CRISPR RNA). They have been engineered as a single guide RNA (sgRNA) with two critical features: a sequence at the 5′ side, that determines the DNA target site by base pairing, and a duplex RNA structure at the 3′ side that binds to Cas9 [40]. This apparatus resulted to be simple because introducing a change in the guide sequence of the sgRNA induces Cas9 to target any DNA sequence of interest [10, 40].

The targeted sequence recognition has two key elements: the protospacer sequence, which has to be complementary to the 5′ -end 20-nt sequence of crRNA, and the presence of an essential short sequence, named protospacer adjacent motif (PAM) bound by Cas9 [40]. The CRISPR/Cas9 system will be able to cut and to introduce a DSB after these two conditions occur: the protospacer pairs to 5′ -end 20-nt sequence and the bound between Cas9 and the PAM sequence [41]. PAM is critical for initial DNA binding, as some studies showed that Cas9 does not recognize target sequences fully complementary to the guide RNA in the absence of PAM [41].

After the system has acted, DNA repair machinery works with the aim to fix the induced DSB catalyzing NHEJ or HDR [41]. The mechanism of CRISPR/Cas9-mediated genome targeting includes conformational rearrangements, the first upon binding to the guide RNA and the second after the association with a target double-stranded DNA. Indeed, Cas9 architectures define two lobes harboring two nucleic acid clefts and undergoing guide RNA-induced reorientation in order to form a central channel where DNA substrates are bound [42, 43].

Despite that most of these knowledges about CRISPR/Cas9 have their origin in microbiological studies, in 2013, it was clear that this system could be efficiently applicable to edit the genome of human cells [40]. In the last few years, RNA-programmable *S. Pyogenes* Cas9-mediated gene editing has been applied to various human cells, including embryonic stem cells [44, 45], with different goals: the precise reproduction of tumor-associated translocations, the analysis of gene functions through loss-of-function genetic screening, and above all the correction of genetic mutations responsible for inherited disorders [46–49].

In light of the operating mechanism, it is possible to assume that CRISPR/Cas9 is the most easy-to-use and cost-effective technology, because it requires only a change in the guide RNA sequence to modify the target site [40]. Finally, to make the best use of this gene editing system, it is necessary to design the right sgRNA, choosing the most suitable sgRNA design tools, and to find out potential off-target sites, improving sgRNA specificity [37]. The off-target predominantly occurs at sites bearing a PAM and partially complementary to the guide RNA sequence [37]. However, over the years, many efforts have been focused on the enhancement on the system to reduce off-targets [50, 51]. These efforts have been focused to develop a nickase version of Cas9 (D10A mutant) directed by paired guide RNAs or an engineered Cas9 nuclease variants with enhanced specificity (eSpCas9). Other advances have been oriented towards the development of a CRISPR/Cas9 capable to execute a genome edit without the need of DSBs [52].

The most recent successful attempt to improve this system derives from the prime editing, a versatile and precise genome editing method that uses a catalytically impaired Cas9 fused to an engineered reverse transcriptase, programmed with a Prime Editing Guide RNA (pegRNA) [53]. The special feature lies in a pegRNA that both specifies the target site and encodes the desired edit, thanks to its extension from which it enables the system to copy the genetic information directly [53]. Anzalone et al. reported the potential of this innovative tool to practice a gene editing without DSBs or donor DNA, performing more than 175 edits in human cells, with the plus side to have much lower off-target activity than Cas9. Prime editing was tested on the mutations responsible for sickle cell disease (OMIM #603903) and Tay Sachs disease (OMIM #272800). Moreover, it has been evaluated for the introduction of a protective variant in *PRNP* gene in HEK cells, the insertion of a transversion in *DNMT1* gene in mouse primary cortical neurons, and also for a comparison between prime editing and HDR in four human cell lines (HEK293T, K562, U2OS, and HeLa). The results highlight the good efficiency of the prime editing and its ability to expand the purposes of the genome editing, correcting a high percentage of known genetic variants associated with human disorders, with a lower degree of off-target and fewer byproducts than HDR [53].

## Overview of CRISPR/Cas9 gene editing in hiPSCs

Reprogramming of somatic cells and genome editing represents two technologies capable to change radically biological and medical research in recent years, reinforcing the role of stem cells in translational medicine. After the design of the correct sgRNA, CRISPR/Cas9 can target a wide number of genomic sequences, through gene knockout or knock-in, gene interference or activation, and other chromosome-related applications, maintaining unchanged the remaining part of the genetic background [54].

Starting from the basic biological studies on hiPSCs, CRISPR/Cas9 system has been used in different ways depending on the purpose of the research as follows:

- Gene knockout is mainly applied to study gene function, because it is the most used implement to establish a connection between a biological event and the upstream molecular mechanism [55].
- Gene knock-in, with the introduction of an exogenous nucleotide sequence, is typically responsible of the identification of specific markers in stem cells research [56].
- Transcription activation or repression: some Cas9 variants (e.g., dCas9, dead Cas9) are deprived of their endonucleolytic activity but maintaining unaltered the ability to generate the gRNA/Cas9 complex. These variants could be fused with transcriptional activator or suppressor, in order to modulate the transcription of endogenous genes [57].
- Genome-wide screening: gRNA libraries provide a large volume of genes for analyzing results through sequencing data collection. While RNA interference (RNAi) libraries knock down gene expression at mRNA level, CRISPR/Cas9 is able to target gene knock-out or transcription inhibitors [58].

These mutations could be evaluated both for basic biological studies on stem cells but also in medical research for disease modelling and drug screening [58].

The first attempts to genetically modify hiPSCs were characterized by the need of an insertion of a floxed resistance cassette. *LoxP* cassette is usually introduced by homologous recombination with the antibiotic gene, but its removal could induce the presence of residual *LoxP* sequences that may induce uncontrolled phenotypes [59].

Even before the setup of a mechanism affording the isolation of single-base genome-edited hiPSCs without antibiotic selection [60], CRISPR/Cas9 had been used to obtain isogenic cell lines for disease modelling and cell therapy. One of the first study focused on this direction was finalized to design a specific gRNA for the correction of a point mutation in *HBB* locus, responsible for sickle cell disease (OMIM #603903) [61]. The compound was designed with the gRNA, the Cas9 associated with a donor DNA template containing the WT HBB DNA, and a selection cassette subsequently removed. The authors differentiated both the corrected and parental hiPSCs into erythrocytes, demonstrating the production of HBB protein from the corrected allele in the erythrocytes derived from gene-edited hiPSCs [61]. The importance of the availability of an isogenic cells line is closely related to the chance to find out the role of a particular gene and thus the consequences of its possible mutation, as described in a recent study [62]. In this paper is reported the correction of *ATM* mutations in Ataxia-Telangiectasia (OMIM #208900) patient-derived hiPSCs through the application of CRISPR/Cas9 approach [62]. Gene corrected hiPSCs showed the improvements reached after gene editing: the restoration of DNA damage and oxidative stress response [62]. Along with the generation of isogenic cell lines, CRISPR/Cas9 technology could be also applied to WT-hiPSCs in order to generate the specific mutation responsible for the investigated pathology. One example has been published very recently to model the autosomic dominant polycystic kidney disease (ADPKD, OMIM #613095) [63]. The study started from hiPSCs derived from healthy individuals where the CRISPR/Cas9-mediated knocked out of the *PKD2* gene was performed [63]. The same approach has been followed for the introduction of a patient specific point mutation in *MEN1* gene into WT cell line, including a donor oligonucleotide carrying the mutation [64]. Here, the association between the gene editing and the hiPSCs helped to explain the molecular differences in hypoglycemic phenotype showed in two patients carrying the same mutation [64].

## Application of CRISPR/Cas9 to hiPSCs for the discovery of new therapeutic strategies

Given the potential of the multiple applications of CRISPR/Cas9 to hiPSCs, their combination can be considered an opportunity for the development of novel therapeutic strategies.

The main ways to get CRISPR/Cas9 and hiPSCs closer to pharmaceutical research are treated below and summarized in Table 2.

**Table 2:** Summary of the applications of CRISPR/Cas9 on hiPSCs for the identifications of therapeutic strategies

	Research field	hiPSCs-derived cells	CRISPR/Cas9 gene editing	Outcome	Ref.
**Drug development**	Evaluation of PEPT1-mediated intestinal absorption	Intestinal epithelial-like cells	Peptide transporter 1 (PEPT1)-knock-out iPSCs	Setting the basis for the development of peptide and peptide-mimetic drugs as possible substrates of PEPT1	[65]
	Multiple-system atrophy (OMIM #146500)	Neurons	Correction of *COQ2* mutation	Identification of Q10 as possible therapeutic target	[66]
**Monitoring of protein dosage**	FOXG1 syndrome	Interneurons	Tag *FOXG1* gene with small molecule-assisted shut-off (sMASh)	Demonstration of FOXG1 dose-control	[67]
**Gene therapy**	Beta-thalassemia (OMIM #613985)	Hematopoietic stem cells	Correction of *HBB* mutation	Corrected-hematopoietic stem cells transplantation as therapeutic strategy	[68, 69]
	Recessive dystrophic epidermolysis bullosa (OMIM #226600)	Keratinocytes and fibroblasts	Correction of *COL7A1* mutation	Restoration of the regular collagen type VII expression	[70]
	Duchenne muscular dystrophy (OMIM #310200)	Skeletal muscle cells	*DMD* exon 44 knock-in	Restoration of full protein coding-region	[71]
**Drug screening**	mtDNA depletion syndrome (OMIM #251880)	Hepatocytes	Inducing *DGUOK* knock-out	Identification of compound able to restore mithocondrial function	[72]
	Alzheimer’s disease (OMIM #104300)	Neurons	Correction of *PSEN1* G384A mutation	Identification of a synergistic combination of bromocriptine, cromolyn and topiramate as an anti-Aβ cocktail	[73]
**Immune response strategy**	HIV infection	Macrophages	Introduction of 32bp-depletion in *CCR5* gene	Generation of immune cells resistant to HIV-infection	[74]
	HIV infection	Monocytes/macrophages	Engineer hiPSCs to express a CRISPR/Cas9 system directed against the reverse-transcribed products of the viral RNA genome	Stable expression of HIV-targeted CRISPR/Cas9 in hiPSCs-derived reservoir cells	[75]
	SARS-Cov-2 infection	Pneumocytes type II	Regulation of genes involved in viral infection	Building a cell platform to test the capacity of candidate antiviral compounds	[76]
	Solid tumors	Natural killer	hiPSCs were edited with CRISPR/Cas9 to repress *ADAM17* expression	Obtaining natural killer cells directed against tumor cells	[77]

### Drug development

Through genetic manipulation applied to hiPSCs, it is possible to introduce particular genetic changes in the early phase of drug discovery process that could be useful for the development of a particular compound or to choose the perfect drug candidate. Recently, Kawai et al. [65] have published their experiments about the use of CRISPR/Cas9 to induce peptide transporter 1 (PEPT1)-knock-out hiPSCs, differentiating them into intestinal epithelial-like cells (IECs). The aim of this study was to evaluate the PEPT1-mediated intestinal absorption, in order to set the basis for the development of peptide and peptide-mimetic drugs as possible substrates of PEPT1 [65]. Thus, the use of hiPSCs found its emplacement into pharmacokinetic testing. They demonstrated that PEPT1 expression levels in hiPSCs-IECs were similar to those in human adult small intestine and that these cells exhibited PEPT1 activity. For the first time, CRISPR/Cas9 was used to deplete a particular glutamate present in exon 21, required for PEPT1 transport ability. The result was the constitution of a PEPT1-KO-hiPSCs-IECs line, which can be employed in highly specific transporter assay for the evaluation of PEPT1 substrates, without the use of inhibitors [65].

Another step of drug development is the identification of specific therapeutic targets involving the use of hiPSCs and CRISPR/Cas9 gene editing. Their assembling was implemented in the research about a particular neurodegenerative disease named multiple-system atrophy (MSA, OMIM #146500) [66]. MSA is characterized by autonomic failure with different classifications based on the involvement of parkinsonism, cerebellar ataxia, and pyramidal dysfunction. Nakamoto et al. [66], with the aid of hiPSCs-patient-derived neurons, studied a form of MSA related to a functionally impaired variant of *COQ2* gene, associated with a reduction in Q10 biosyntesis. By using the gene correction mediated by CRISPR/Cas9, it was possible to reverse the resulting functional deficiencies in mitochondrial respiration and antioxidative system as well as the increased apoptosis. Hence, this study relates neuron dysfunctions with the decreased coenzyme Q10, thus assuming that a supplementation of Q10 could represent a useful option for MSA therapy [66].

### Monitoring of protein dosage

Monitoring the amount of particular proteins is one of the main purposes in the investigations of disorders characterized by the accumulation of some products. After all, there are genetic alterations inducing half-loss, functional impairment, and de novo gain of function which are responsible for abnormal protein dosage [78, 79]. One example is the unusual storage of key regulators in developmental disorders, as in FOXG1 syndrome (OMIM #613454). Forkhead transcription factor1 (FOXG1) is variably expressed at early stage of brain development [80]. It has been already demonstrated that human deletions or missense mutations on one allele of *FOXG1* cause severe neurodevelopmental disorders named FOXG1 syndrome, with many phenotypic manifestations including autism spectrum disorder, epilepsy, microcephaly, and severe intellectual disability [81–83]. Zhu et al. [67] for the first time tried to control FOXG1 dosage by combining three technologies: CRISPR/Cas9, small molecule-assisted shut-off (SMASh), and hiPSCs. This novel combination allows to better manage the difficulties in dosage control usually met when traditional knock-out or knock-down strategies are used. Upon the administration of small molecules (protease inhibitors), SMASh technology is able to alter the post-translational amount of proteins in a precise and reversible manner, thanks to the presence of self-removing degrons [67]. With CRISPR/Cas9, the authors tagged the targeted gene with a SMASh in hPSCs-derived interneurons. Through this architecture, they have been skilled to monitor how FOXG1 dosage affects the generation of GABAergic interneurons, which might explain the variable clinical manifestations of FOXG1 syndrome. The authors tuned this method in order to reproduce a disease model, because controlling protein dosage by drug-induced degradation or stabilization makes it easy to study protein function [67]. However, this could find interesting applications in pharmaceutical purposes screening drugs with the aim to restore the normal protein quantity, in order to decrease or remove the severe clinical manifestations of syndromes due to alterations in protein amount.

### Gene therapy

One of the main applications derived by the fusion of hiPSCs with CRISPR/Cas9 is gene editing for cell therapy. In fact, hiPSCs allow to take patient cells where genome edit is practicable, and then it is feasible to use them for autologous cell transplant. In 2015, it was demonstrated that the *HBB* gene correction with CRISPR/Cas9 of beta-thalassemia (OMIM #613985) patient-specific hiPSCs, and their derived hematopoietic stem cells transplantation, offers an ideal therapeutic solution for treating the disease [68, 69].

Progress have been also done in studying the recessive dystrophic epidermolysis bullosa (RDEB, OMIM #226600), a severe inherited skin disorder caused by mutations in *COL7A1* gene [84]. Jackòw et al. [70] used hiPSCs derived from RDBE patients and corrected them with CRISPR/Cas9. Three-dimensional skin equivalents (HSEs) were generated from gene-corrected hiPSCs, differentiated into keratinocytes and fibroblasts, and then grafted into immunodeficient mice [70]. Animals showed normal expression of type VII collagen. Therefore, this laid the foundation for future clinical applications of innovative autologous stem cell-based therapies for RDBE [70].

Another study has been focused on the correction of the Duchenne muscular dystrophy (DMD)-causing mutation with TALENs and CRISPR/Cas9 in patient-derived hiPSCs [71]. DMD (OMIM #310200) is a severe muscular degenerative disease caused by loss of function mutations in the dystrophin gene (*DMD*) located on chromosome X [71]. Patient myoblasts had been previously used for restoration of the dystrophin protein, but their clonal expansion required the immortalization process mediated by oncogene such as hTERT [71, 85]. Instead, hiPSCs can be isolated from patients maintaining their pluripotency and self-renewal capacity without any further procedures. In this study [71], the authors tried to perform dystrophin correction using three different methods: disruption of the splicing acceptor to skip exon, introduction of small indels to modulate the protein reading frame, and knock-in of the missing exon 44 to restore the full protein coding-region [71]. The results showed that all of these approaches were beneficial, but only the knock-in approach restored the full-length dystrophin protein [71]. The investigations about the Duchenne disease have sustained important progress in order to reduce the probability of off-targets mutagenesis and immunogenicity, generally due to the prolonged expression of the CRISPR/Cas9. Very recently, Gee et al. [86] have developed an extracellular nanovesicle-based ribonucleoprotein delivery system named NanoMEDIC. This is developed on chemical dimerization and viral RNA packaging signal to recruit Cas9 protein and sgRNA into nanovesicles [86]. They have tested this device in different cell types, among others in skeletal muscle cells derived from DMD-hiPSCs. The system reached over 90% exon skipping efficiencies. This innovative technique, as in vivo experiments have demonstrated, seems to be promising for DMD genome editing therapy [86].

### Drug screening

The potential and availability of genome editing and reprogramming equipment place at disposal of researchers a valid instrument for drug screening, especially when there is a lack to access to patient samples. After gene editing, cells can be differentiated into specific cell types, target of disease, and a screening of different compounds can be performed, in order to identify those able to ameliorate the disease phenotype. For example, drug screening resulted to be the best way for the identification of the ideal compound to fight the mtDNA depletion syndrome (MTDPS3, OMIM #251880) caused by the deficiency of DGUOK, a mitochondrial kinase responsible for the phosphorylation of purine deoxyribonucleosides [87]. The liver is the main organ involved in this disease [72]; thus, authors derived hepatocytes from WT-hiPSCs, after inducing *DGUOK* knockout through CRISPR/Cas9. These cells recapitulated the mitochondrial dysfunction associated with the livers of MTDPS3 patients and consequently were used as platform to identify drugs that could improve mitochondrial function and ATP production [87]. The authors used the SPECTRUM collection library, which contains about 1300 drugs approved for human use in the USA, Europe, and Japan. ATP levels were used to identify compounds that increase cellular energy production. Among the drug examined, NAD was the one that reproducibly increases ATP levels and the expression of all mitochondrial-encoded electron transport chain genes evaluated in this screening [87]. Hence, a possible treatment with NAD could represent a valid therapeutic option. This is one example of how the conjunction of reprogramming and gene editing can provide a high-throughput test system for screening potential therapeutic preparations with different activity spectra.

### Immune response strategy

As previously described, CRISPR/Cas9 was born as immune response in some bacteria for virus removal. This concept has been applied to HIV research, because the actually available therapeutic strategies are not able to totally eradicate the virus from the body and to date many studies are trying to apply genome editing to fight this infection [74]. Concerning the possible application of hiPSCs in this field of medical research, *Liao* and coll [75]. tried to induce into hiPSCs an antiviral defense system similar to those of bacteria and archaea. They engineered hiPSCs to express a CRISPR/Cas9 system directed against the reverse-transcribed products of the viral RNA genome. The result was the production of hiPSCs which stably expressed HIV-targeted CRISPR/Cas9. These cells can be differentiated into HIV reservoir cells, maintaining a long-term resistance to the virus. So this can be defined an innovative therapeutic strategy against viral infections [75].

Concerning the possible applications of CRISPR/Cas9 and hiPSCs in antiviral response, this apparatus can find an employment in the more current than ever SARS-Cov-2 research. The driven idea is the building of a testing platform that mimics a human lung, differentiating WT-hiPSCs into pneumocytes type II [88] and treating them with pseudoviruses able to mimic SARS-Cov-2 infection [89]. Then, gene editing can be employed for the repression or the upregulation of genes involved in virus entrance and activity but also for the introduction of known polymorphisms that could protect or predispose to virus infection [90]. Also, edited cells can also be used to test the capacity of a number of candidate compounds to fight the infections. Very recently, a flexible and efficient approach to target virus RNA through CRISPR/Cas9 action has been implemented; it can be used specifically for SARS-CoV-2 RNA genome, limiting its ability to reproduce [76]. This represents a great opportunity to fight viruses that have the potential to evolve and develop resistance rapidly [76].

Another medical research field, which required the stimulation of the immune response, is oncology. CRISPR/Cas9 in cancer research is already widely used, because the ability to identify and correct such mutations, but also the discovery of molecular targets, are important aims of cancer treatment [91–93]. However, oncology projects exploiting the combination between CRISPR/Cas9 and hiPSCs are scarce. An interesting research has been published in 2016 [77], when a group of researchers tried to improve NK cell-mediated killing solid tumors, engineering them to express a more stable form of CD16a. This protein has a key function in the elimination of cells opsonized by antibodies through the Antibody-Dependent Cell-mediated Cytotoxicity (ADCC). NK cells activity could be hampered by the expression of ADAM17, a metalloprotease that mediated the proteolytic cleavage of CD16a. Thus, hiPSCs were edited with CRISPR/Cas9 to repress ADAM17 expression and soon after also engineered to produce the cleavage-resistant CD16a (S197P). These cells have been differentiated into natural killer ones directed against tumor cells, representing an approach for allogenic cell-based immunotherapies [77].

## Conclusions and future challenges

The reprogramming technology with the resulting possibility to use patient-derived pluripotent stem cells has implemented medical research. This breakthrough has undergone a further advancement after the discovery of CRISPR/Cas9 genome editing system. These two tools have a multitude of roles both in basic biological and translational research, with a particular involvement in pharmacological fields and drug development.

However, many challenges have to be overcome. In particular, it is necessary to improve reprogramming mechanism efficiency and to set up standard protocols, avoiding incomplete reprogramming and the onset of de novo mutations.

The revolution started by the introduction of such advanced tool for gene editing based on a single protein makes difficult to image a system simpler than CRISPR/Cas9. Anyway, its enhancement is required in order to reduce off-targets effects of genome editing and to build novel strategies for CRISPR/Cas9 delivery into cells, without using virus carriers, unsuitable for clinical application. It would also be appropriate to facilitate the selection and expansion of hiPSCs-CRISPR/Cas9-corrected clones, which are often laborious and time consuming.

In addition, there is the need to evaluate the safety aspects for human trials, both for the application of CRISPR/Cas9 and hiPSCs. Another perspective is to improve target accuracy, with the help of pluripotent stem cells, which can be easily translated to preclinical and clinical studies, enhancing the clinical trial success rate.

Working on these points could have important implications in drug development process, reducing time and cost-effects.

## Data Availability

Not applicable

## Abbreviations

iPSCs: Induced pluripotent stem cells
hiPSCs: Human-induced pluripotent stem cells
CRISPR/Cas9: Clustered, regularly interspaced, short palindromic repeats/CRISPR-associated protein
WT: Wild type
FD: Familial dysautonomia
IKBKAP8: IkB kinase complex-associated protein
ALS: Amyotrophic lateral sclerosis
FDA: Food and Drug Administration
CML: Chronic myelogenous leukemia
OOC: Organ-on-a-chip
HTS: High-throughput screening
SFEBs: Serum-free embryoid bodies
HR: Homologous recombination
DSB: Double-strand break
ZFNs: Zinc finger nucleases
TALENs: Transcription activator-like effector nucleases
NHEJ: Non homologous end joining
gRNA: Guide RNA
sgRNA: Single guide RNA
crRNA: CRISPR RNA
tracrRNA: Transactivating crRNA
PAM: Protospacer adjacent motif
pegRNA: Prime editing guide RNA
RNAi: RNA interference
ADPKD: Autosomic dominant polycystic kidney disease
IECs: Epithelial-like cells
PEPT1: Peptide transporter 1
MSA: Multiple-system atrophy
FOXG1: Forkhead transcription factor1
SMASh: Small molecule-assisted shut-off
RDBE: Recessive dystrophic epidermolysis bullosa
DMD: Duchenne muscular dystrophy
MTDPS3: mtDNA depletion syndrome
